# Rethinking knowledge systems in psychology: addressing epistemic hegemony and systemic obstacles in climate change studies

**DOI:** 10.3389/fpsyg.2025.1533802

**Published:** 2025-04-02

**Authors:** Mudassar Aziz, Gulnaz Anjum

**Affiliations:** ^1^Department of Psychology, University of Limerick, Limerick, Ireland; ^2^Department of Psychology, University of Oslo, Oslo, Norway

**Keywords:** climate psychology, western epistemologies, indigenous knowledge, decolonization, methodological pluralism, climate adaptation, participatory research

## Abstract

Climate psychology has emerged as a critical field examining how individuals and societies perceive, respond to, and engage with the climate crisis. However, the discipline remains deeply influenced by Western epistemologies, which privilege individualistic, anthropocentric, and positivist approaches to knowledge production. This perspective paper critically examines how Western bias shapes the theoretical frameworks, methodological approaches, and policy implications within climate psychology, often to the exclusion of non-Western epistemologies, particularly those from Indigenous and Global South communities. We argue that dominant Western paradigms, rooted in individualism, cognitive-behavioral models, and human-exceptionalist perspectives, constrain the field's ability to fully capture the complex, relational, and context-specific ways in which diverse populations engage with climate change. Moreover, the overreliance on quantitative and experimental methodologies systematically marginalizes Indigenous methodologies, such as storytelling, relational worldviews, and participatory research approaches, thereby limiting the inclusivity and ecological validity of climate psychology research. To address these limitations, we propose a decolonial approach to climate psychology, advocating for the integration of Indigenous epistemologies, pluralistic methodologies, and equitable research collaborations. By diversifying epistemic foundations and methodological tools, climate psychology can move beyond its Western biases, leading to more culturally responsive research and more effective and just climate interventions. This paper calls for a fundamental reorientation in climate psychology, one that values epistemic diversity as essential for addressing the multifaceted human dimensions of climate change.

## 1 Introduction

### 1.1 Academic dominance in psychology

Academic dominance refers to the structural and systemic privileging of Western, particularly Eurocentric, perspectives in scholarly communication, research methodologies, and education. This epistemological bias marginalizes indigenous and non-Western knowledge systems, reinforcing Western-centric theories as universal while disregarding the cultural and historical contexts from which they emerge (de Sousa Santos, 2007; Dei, 2000). Academic gatekeeping is evident in journal editorial policies, funding allocations, and citation practices, all of which favor research aligned with Western academic priorities (Arnett, 2008; Connell, 2007). The predominance of English as the primary language of academic discourse further limits the visibility of non-English research, excluding diverse perspectives from global scholarship (Phiri et al., 2023; Adams et al., 2015). The linguistic hegemony of English exacerbates these disparities, creating barriers to knowledge production and dissemination. Non-Western psychological constructs are frequently lost in translation or misrepresented within Western theoretical frameworks, further entrenching Anglo-centric dominance in the field.

In psychology, this bias manifests in both research and clinical practice, shaping a homogenized portrayal of human behavior based on Western norms. Western psychological theories dominate curricula and empirical research, sidelining indigenous psychologies and alternative epistemologies (Allwood and Berry, 2006; Henrich et al., 2010; Teo, 2015a,b). Editorial biases in psychology journals further reinforce this hierarchy, privileging studies that align with Western conceptual frameworks and restricting the inclusion of diverse perspectives (Arnett, 2008). The issue extends to funding, with Western institutions primarily supporting research that aligns with their interests, limiting the exploration of regionally relevant psychological phenomena (Connell, 2007). Intellectual migration also contributes to this imbalance, as scholars from the Global South often relocate to the Global North, exacerbating the unidirectional flow of knowledge. In climate psychology, for instance, Western-centric models frequently overlook the contributions of indigenous and non-Western communities, despite their disproportionate exposure to climate change (Adger et al., 2013). Similarly, health psychology often disregards holistic wellness models from non-Western cultures in favor of Western behavioral frameworks (Kirmayer, 2012).

Psychology's intellectual dominance is rooted in the overrepresentation of Western paradigms, methodologies, and epistemologies, leading to a narrow, culturally specific understanding of human cognition and behavior. The discipline is disproportionately shaped by research from Western, Educated, Industrialized, Rich, and Democratic (WEIRD) societies, which are often treated as universal standards (Henrich et al., 2010). This bias manifests in two key ways: first, psychological norms derived from WEIRD populations are positioned as global benchmarks, implicitly casting non-Western variations as deviations (Markus and Kitayama, 1991). Second, researchers from the Global South, despite providing critical cultural and linguistic insights, are often under-recognized and undercompensated, reinforcing academic inequities (Crane, 2010; Anjum and Aziz, 2024).

Cross-cultural collaborations often reflect these power imbalances, with Western institutions setting research agendas and marginalizing local priorities (Mignolo, 2009). The resource gap further restricts opportunities for non-Western scholars, limiting their academic recognition and career advancement. This asymmetry also affects the epistemological foundations of psychology, as dominant Western theories, often rooted in individualism, fail to capture relational and collectivist perspectives prevalent in other cultures (Christopher et al., 2014; Adams and Salter, 2007). Moreover, psychology's preference for positivist, quantitative methodologies often overlook complex, culturally embedded psychological phenomena, leading to reductive interpretations and interventions (Bhatia and Priya, 2018; Bhatia, 2002; Dudgeon et al., 2000).

Psychology must integrate indigenous knowledge, adopt participatory research methods, and reform curricula to reflect global psychological diversity (Reason and Bradbury, 2008). Ethical considerations in cross-cultural research must also be prioritized to ensure equitable collaboration and representation (Marshall and Koenig, 2004). Expanding interdisciplinary partnerships and revising publication practices will foster a more inclusive and culturally attuned discipline, ultimately broadening psychology's global applicability and impact. Addressing these issues requires a shift toward more inclusive research paradigms, equitable knowledge-sharing practices, and a re-evaluation of psychology's foundational assumptions to better reflect the full spectrum of human experience.

Having established the broader issue of Western dominance in psychology, this chapter now transitions to a focused examination of biases in climate psychology. While mainstream psychology has long been criticized for its overreliance on Western paradigms, these biases are particularly consequential in climate psychology, where cultural, historical, and socio-political contexts shape both experiences of and responses to climate change. The influence of Western epistemologies in climate psychology manifests in theoretical frameworks, methodological approaches, and policy applications, often marginalizing indigenous and non-Western ways of knowing. From this point forward, the paper critically explores five key areas: the dominance of Western theoretical paradigms, methodological exclusions of non-Western perspectives, empirical case studies demonstrating these biases, the impact of epistemic dominance on climate change policies, and potential strategies for fostering greater epistemic diversity. By highlighting these dimensions, the discussion underscores the need for an inclusive and pluralistic approach that acknowledges the global and intersectional nature of climate change and its psychological consequences.

### 1.2 Hegemony of western epistemologies and climate change psychology

Climate change psychology examines how people perceive, feel, and act regarding the climate crisis. However, much of this research is rooted in Western epistemologies—the ways of knowing and understanding prevalent in Western societies. Psychological science has long been dominated by studies on WEIRD (Western, Educated, Industrialized, Rich, and Democratic) populations, leading to theories and methods that assume Western norms as universal (Kim et al., 2023; Henrich et al., 2010). One such Western paradigm is human exceptionalist (HE) thinking, which conceptualizes humans as distinct from and independent of the natural world. HE is particularly pervasive in WEIRD societies and has profound implications for climate change psychology. It shapes how individuals perceive their relationship with nature, influencing their attitudes toward environmental crises, conservation, and climate policy.

Kim et al. (2023) argue that HE fosters a cognitive framework in which humans are seen as separate from ecological constraints, reducing the urgency of pro environmental behavior and climate mitigation efforts. Empirical studies show that HE is negatively associated with concern for climate change, willingness to engage in conservation, and recognition of human dependence on ecosystems. This cognitive bias may contribute to the slow response to the climate crisis in WEIRD societies, where policies often reflect an implicit assumption that technology and human ingenuity can override ecological limits. Moreover, HE contrasts with many Indigenous and non-Western epistemologies that emphasize interconnectedness between humans and nature.

The dominance of HE in Western climate change psychology exemplifies how mainstream research methodologies may overlook alternative ways of understanding environmental challenges. Theoretical models based on HE may fail to capture how non-WEIRD populations conceptualize human—nature relationships, leading to incomplete or biased psychological theories of climate change perception and action. To address this limitation, climate psychology must integrate non-Western epistemologies that emphasize relational perspectives, reciprocity with nature, and collective responsibility. Research suggests that interventions highlighting the interconnectedness between humans and ecosystems—such as framing climate impacts locally or encouraging systems thinking—can help reduce HE thinking and foster more sustainable attitudes (Kim et al., 2023).

By critically examining the influence of Western paradigms such as HE, climate psychology can move toward a more inclusive and globally relevant understanding of environmental cognition and behavior. Recognizing and integrating non-Western perspectives into research methodologies and climate policies will be essential for addressing the climate crisis in a way that is both culturally sensitive and ecologically informed.

Climate change psychology has emerged as a critical field for understanding human responses to the climate crisis; however, much of its research remains rooted in Western epistemologies, limiting its global applicability. This paper critically examines how Western paradigms shape climate psychology research, highlighting the need for more inclusive frameworks. First, we explore the theoretical foundations of climate psychology, focusing on how Western individualism, cognitive-behavioral models, and human-nature dualism have influenced the discipline. Second, we assess the methodological limitations of Western-centric research, including its reliance on quantitative methods while overlooking Indigenous and community-based approaches. Third, we present empirical case studies that demonstrate how Western biases shape research outcomes, often misrepresenting or marginalizing the perspectives of non-Western and Indigenous communities. Fourth, we analyze the impact of Western epistemologies on climate change policy, revealing how Western-centric psychological models shape public engagement strategies, adaptation planning, and international climate negotiations in ways that may not align with diverse cultural understandings. Finally, we propose solutions for integrating non-Western epistemologies, including methodological pluralism, Indigenous collaboration, participatory action research, and policy recommendations that embrace epistemic diversity. By broadening the epistemological scope of climate psychology, this research aims to foster a more globally relevant and socially just understanding of the psychological dimensions of climate change.

#### 1.2.1 Theoretical framing: western paradigms in climate change psychology

Western psychological paradigms provide the dominant lens through which climate change is understood in mainstream research. One key influence is individualism, a value emphasizing personal agency and independence that is common in Western cultures. As a result, climate psychology often focuses on individual attitudes and behaviors—for example, studying personal risk perception, eco-anxiety, or “green” consumer choices—assuming that individuals are the primary unit of change. In contrast, more collectivist cultures stress community and relational responsibilities. Research indicates that individualist orientations (more typical in the West) can actually dampen climate action willingness compared to collectivist orientations (Xiang et al., 2019). In a cross-cultural study, participants with strong individualist values were less likely to take climate-friendly action than those with collectivist values, highlighting how Western individualism may bias the understanding of what motivates climate action (Xiang et al., 2019). Western frameworks may therefore overlook the communal and interdependent motivations that drive environmental engagement in other societies.

Another Western paradigm shaping theoretical framing is the way humans' relationship to nature is conceptualized. Western thought has often been characterized by an anthropocentric or *human-exceptionalist* worldview—the idea that humans are separate from and superior to nature (Kim et al., 2023). This perspective, rooted in centuries of Western philosophy and science, promotes a sharp boundary between humans and the rest of the natural world. In climate psychology, this can lead to theories that treat “the environment” as an external object or resource for humans, rather than as a web of relations that include humans. Non-Western epistemologies frequently espouse more holistic human—nature relationships. For instance, many Indigenous worldviews see humans as deeply embedded in ecological systems, emphasizing reciprocity and kinship with the natural environment (David, 2024). Western psychology's dominant models—such as cognitive-behavioral theories—tend to focus on internal mental processes (e.g., risk cognition and personal efficacy) while downplaying spiritual, emotional, and relational dimensions of climate responses that are prominent in other cultural paradigms. Critics note that mainstream climate psychology often relies on a one-sided view of human nature that foregrounds individual cognitive barriers and rational choice, reflecting Western intellectual traditions (Schmitt et al., 2020). This can exclude rich understandings from non-Western traditions, such as the Ubuntu philosophy in parts of Africa, which frames individual well-being as inseparable from community and environment (David, 2024).

Risk perception in climate change psychology is deeply influenced by cultural perspectives. Predominant Western frameworks often prioritize material and immediate risks, overlooking the complex spiritual or relational perspectives significant in many non-Western and indigenous cultures (Ojala, 2012; Leiserowitz, 2006). This narrow view can lead to culturally insensitive interventions that fail to align with local values or leverage traditional ecological knowledge, thus diminishing their effectiveness (Adger et al., 2013). Incorporating a broader spectrum of risk perceptions, informed by interdisciplinary research and indigenous knowledge systems, is critical for designing culturally sensitive and effective interventions (Slovic, 2010; Crate and Nuttall, 2016; Cameron, 2012; Whyte, 2017b).

In the study of climate psychology, research has predominantly concentrated on Western populations, often overlooking the insights and experiences of indigenous communities and societies outside the Western sphere. These groups are not only more susceptible to the impacts of climate change but also hold invaluable ecological knowledge crucial for developing adaptive strategies (Adger et al., 2013). Recent research has started to focus on the cultural and social dimensions of climate change, alongside the traditionally studied physical, biological, and economic aspects. This body of work highlights the importance of “places” as locales imbued with deep cultural significance and advocates for the consideration of the potential irreversible loss of such places in climate change policymaking, emphasizing principles of fairness and the acknowledgment of community identities (Adger et al., 2013).

**Figure 1:**
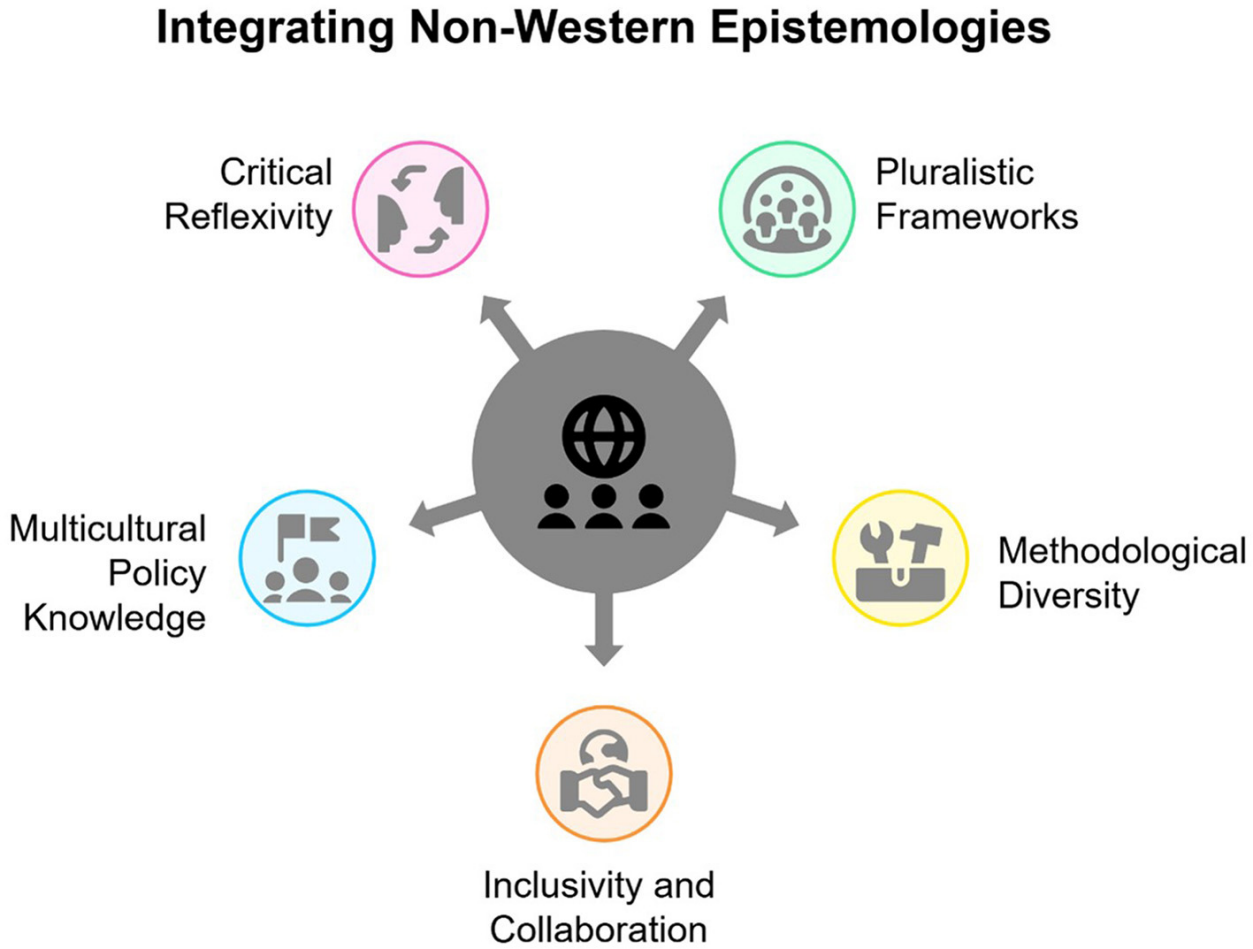
Proposed solutions: integrating non-western epistemologies in climate psychology.

The inclusion of indigenous and local wisdom into psychological frameworks for climate adaptation and ecological preservation is emerging as a vital approach. This strategy acknowledges the significant role that local and indigenous communities play in environmental stewardship, despite being disproportionately affected by climate change (Adger et al., 2013). Indigenous knowledge systems, with their deep-rooted ecological insights, coping mechanisms, and adaptive strategies developed through centuries of direct interaction with their environments, provide a rich resource for understanding and addressing climate change (Berkes et al., 2000). Figure 1 summarizes the strategies proposed by this paper.

##### 1.2.1.1 Incorporation of diverse constructs

Expanding the range of psychological perspectives within climate psychology is essential for enhancing the field's relevance and applicability across various cultural contexts. A key strategy for achieving this expansion is the incorporation or reinterpretation of psychological concepts grounded in non-Western viewpoints. Clayton and Myers (2015) highlight the value of “ecocentrism,” a concept derived from indigenous philosophies, as an alternative to the predominantly anthropocentric views in Western environmental thought (Clayton and Myers, 2015; Norgaard, 2011). Ecocentrism fosters a more comprehensive understanding of the relationship between humans and nature, emphasizing the interconnectedness of all elements within an ecosystem, in contrast to viewing humans as separate or above nature (Berkes, 2017). Indigenous cultures often embody principles that recognize the interdependence of community well-being and environmental health, offering rich, conceptual contributions to climate psychology that move beyond the individual-centric approaches prevalent in Western models (Whyte, 2017a; Ojala, 2012). Research within climate psychology has largely been shaped by Western traditions, limiting the discipline's scope and the universal applicability of its constructs. Constructs like “environmental identity” and “pro-environmental behavior” are often rooted in individualistic frameworks, focusing on personal beliefs and actions rather than collective or communal practices (Clayton et al., 2016; Gifford, 2011; Gifford and Nilsson, 2014). Such perspectives may not fully represent the environmental engagement of people from cultures with a more community-oriented outlook, thus restricting the global relevance of the field.

In many cultures outside the Western paradigm, environmental engagement is viewed as a collective responsibility, deeply integrated with social norms, spiritual beliefs, and communal activities, making the individualistic measures of environmental behavior less relevant (Jessen et al., 2022). Ignoring these cultural differences can lead to the development of ineffective or culturally insensitive interventions and policies, as strategies based on Western concepts of environmental risk perception might not align with the worldviews of other communities, potentially resulting in disengagement or distrust (Leiserowitz, 2006; Swim et al., 2009). There is a growing recognition of the need to include cross-cultural perspectives in climate psychology research. Adopting constructs that resonate with collectivist values, such as “interdependent self-construal,” could bridge the gap between Western-centric models and the interconnectedness emphasized in many indigenous and Eastern philosophies (Markus and Kitayama, 1991). Engaging with indigenous knowledge systems and local experts in developing psychological concepts and interventions can lead to a more diverse and inclusive approach. This approach requires not just the addition of non-Western perspectives but a fundamental reevaluation of existing frameworks to accommodate a variety of worldviews (Adams et al., 2015). The current dominance of Western-centric theories in climate psychology risks overlooking or misrepresenting the perspectives of a significant portion of the global population. Thus, integrating collective or community-based frameworks into the field is crucial for developing effective and universally applicable interventions.

##### 1.2.1.2 Incorporation of traditional ecological knowledge (TEK)

“Traditional Ecological Knowledge” (TEK) represents a key concept in this integrative approach, encompassing the accumulated ecological understandings, practices, and cultural beliefs of indigenous peoples passed through generations (Salick and Ross, 2009; Berkes, 2017). Research shows that TEK offers detailed insights and unique solutions for contemporary environmental challenges that are often overlooked in mainstream, Western-centric environmental and climate psychology analyses (Nadasdy, 1999).

Methodologically, the integration of indigenous and local insights into psychological research can be advanced through Participatory Action Research (PAR) (Reason and Bradbury, 2008). This method involves community members in the research process, facilitating co-created knowledge and valuing the diverse epistemological contributions of different cultures. It aims to democratize knowledge production by flattening traditional research hierarchies, making it an effective approach for enriching psychological research with indigenous perspectives (Smith, 2012). Psychological frameworks might benefit from incorporating indigenous concepts of “relationality,” enhancing our understanding of human-environment interactions (Kimmerer, 2013). For instance, the Maori concept of “kaitiakitanga,” denoting a kinship-based environmental guardianship, could inform psychological perspectives on environmental stewardship (Kawharu, 2000). However, integrating indigenous and local wisdom necessitates careful attention to avoid appropriation and ensure equitable partnerships, guaranteeing that indigenous peoples are acknowledged as co-authors of their knowledge within academic research (Cochran et al., 2008; Whyte, 2017a).

Incorporating this wisdom into climate psychology can enrich the field's conceptual frameworks and lead to more effective, culturally attuned interventions. This process is part of a broader effort to ensure a more inclusive and holistic approach in psychological science. Climate psychology, in particular, stands to benefit from participatory and co-creative research methods. Such methods not only offer a more equitable and inclusive route to knowledge production but also ensure that the field addresses the unique challenges faced by marginalized and indigenous communities most affected by climate change (Adger et al., 2013).

#### 1.2.2 Methodological limitations of western approaches

Mainstream climate change psychology has been methodologically shaped by Western scientific traditions, often leading to the overlooking of Indigenous and non-Western ways of knowing. Western research methods typically prioritize quantitative data, standardized surveys, laboratory experiments, and other positivist approaches aimed at objectivity. While these methods yield valuable insights, they carry implicit biases about what “valid” knowledge is. Western scientists often assume their approach is neutral and universal, an attitude sometimes termed *scientism*. This assumption can marginalize other knowledge systems. As one analysis notes, many North–South research collaborations fail to recognize diverse worldviews because of the unintentional but prevalent view that Western science is inherently objective and superior (Turner et al., 2024). In practice, this has meant that climate psychology studies rarely incorporate methodologies that fall outside the Western canon, such as oral histories, storytelling, participatory observation, or spiritual interpretations of ecological change that are common in Indigenous research.

A significant limitation is the exclusion of qualitative and community-based methods that carry non-Western epistemologies. Indigenous and other non-Western communities often transmit knowledge through narrative, lived experience, and communal learning. However, traditional climate psychology might consider such qualitative data as anecdotal or less rigorous. For example, the Two-Eyed Seeing approach (Etuaptmumk, from Mi'kmaq tradition) advocates using one eye with the strengths of Indigenous knowledge and the other with Western science, to see more fully (Turner et al., 2024). Yet, few climate psychology studies have embraced such blended frameworks, and most remain siloed in Western methodologies. Similarly, Participatory Action Research (PAR), which involves community members in co-designing research and emphasizes social change, is aligned with many Indigenous approaches but is underutilized in mainstream climate psychology. The dominance of Western methods can also be seen in publication biases: researchers from the Global South (often home to Indigenous communities) are underrepresented in top journals, and Western scholars often study non-Western populations without collaborating with local researchers (Anjum and Aziz, 2025). This “parachute science” means research designs may not respect local ways of knowing (de Vos and Schwartz, 2022). The outcome is an epistemological bias, climate psychology's methods systematically filter out insights that don't fit Western paradigms. In sum, Western methodological dominance from the privileging of quantitative metrics to the marginalization of community-led inquiry, limits the field's ability to fully understand and address climate change through a multicultural lens.

##### 1.2.2.1 Integration of participatory action research (PAR)

Participatory action research (PAR) in climate psychology allows for the exploration of lived experiences and indigenous knowledge systems that are often ignored in dominant climate discourse (Whyte, 2017a). For example, the deep ecological insights from indigenous communities are crucial for a nuanced understanding of climate adaptation and mitigation (Ford et al., 2016). Participatory methodologies foster a co-creation process that can identify locally relevant psychosocial stressors and coping mechanisms, leading to more culturally sensitive psychological interventions (Cunsolo Willox et al., 2013). This collaborative approach not only gives communities ownership over research but also supports sustainable and ethical research practices (Sultana, 2007). Moreover, it encourages an interdisciplinary fusion of knowledge, enhancing the understanding of climate change's psychological impacts through the integration of insights from environmental science, anthropology, sociology, and more (Doppelt, 2016). However, the complexities and potential challenges of participatory and co-creative methods, such as navigating power dynamics and ethical concerns, especially within the context of climate psychology, must be carefully managed (Norgaard, 2011). By embracing these methodologies, climate psychology can play a pivotal role in deconstructing traditional hierarchies of knowledge production, enriching the field with diverse perspectives and creating more nuanced and effective interventions for the psychological dimensions of climate change.

##### 1.2.2.2 Methodological innovation

The preference for quantitative methodologies in climate psychology underscores a broader trend toward methodological uniformity, which raises significant concerns about inclusivity and representation in research. Quantitative methods, often hailed as the benchmark for scientific inquiry, especially in Western contexts, align with a positivist approach that values knowledge acquisition through measurable and universally applicable data (Sale et al., 2002). However, this emphasis can sideline indigenous research techniques or qualitative methods that provide deeper, context-specific insights into human-environment interactions. Indigenous methodologies, which include narrative storytelling, yarning, or community engagement, are fundamentally qualitative and deeply connected to the lived experiences of indigenous peoples. These approaches offer a comprehensive understanding of climate change impacts and psychological aspects, grounded in traditional ecological knowledge (Wilson, 2008; Smith, 2012; Berkes, 2017).

Quantitative research's focus might also neglect the socio-cultural dimensions that qualitative research captures more effectively. Narratives around nature, place attachment, and community resilience to climate change are often deeply embedded in culture and are not easily quantifiable (Vedwan and Rhoades, 2001; Tschakert, 2007). By primarily relying on quantitative methods, there's a risk of overlooking these critical qualitative insights, thus limiting the discipline's methodological diversity. Moreover, an overreliance on quantitative approaches may restrict participatory research opportunities, which aim to democratize the research process by engaging community members directly (Reason and Bradbury, 2008). Participatory methodologies are versatile, allowing for the integration of both quantitative and qualitative data, and are particularly conducive to incorporating indigenous knowledge systems (Cornwall and Jewkes, 1995). While quantitative methods are valued for their generalizability and precision, their predominance can overshadow indigenous and qualitative research methods. This imbalance can detract from a holistic understanding of climate change's psychological impacts, which are inherently context-dependent and culturally specific.

Enhancing climate psychology involves multifaceted efforts to diversify the discipline's methodological and theoretical approaches. Integrating indigenous and local knowledge enriches theoretical frameworks and leads to more culturally attuned interventions (Whyte, 2017a; Berkes, 2017; Trosper, 2009). Additionally, community-based participatory research methods facilitate a more inclusive approach to knowledge creation, rooted in the direct experiences and perspectives of communities (Reason and Bradbury, 2008; Wallerstein and Duran, 2010; Minkler and Wallerstein, 2008). This method engages community members throughout the research process, ensuring that research is relevant and reflective of community needs.

Adopting a pluralistic stance toward epistemology encourages the inclusion of a variety of methodologies and perspectives, moving beyond the limitations of a Western-centric focus (Weintrobe, 2012; Kovach, 2009). By embracing methodological diversity, climate psychology can gain a fuller understanding of varied cultural perceptions and responses to climate change. Thus, the drive toward diversifying climate psychology is not merely academic but a comprehensive initiative to make the field more inclusive, equitable, and effective in confronting the complex challenges posed by climate change.

In essence, addressing methodological uniformity in climate psychology is vital for the discipline's accuracy, relevance, and cultural sensitivity. Embracing a more inclusive and diversified methodological and epistemological approach is crucial for ensuring that psychological knowledge encompasses a broad spectrum of cultural perspectives. This is especially pertinent in specialized areas such as climate psychology, where understanding and interventions must be attuned to diverse cultural realities. Overcoming the deeply rooted methodological biases requires concerted, ongoing efforts to create a discipline that is more inclusive, diverse, and committed to social justice.

#### 1.2.3 Empirical case studies of western bias in climate psychology

Evidence of Western bias in climate psychology research emerges clearly in comparative studies and real-world cases. One illustrative case comes from sub-Saharan Africa: David (2024) examined how Western *scientism*—the overreliance on scientific positivism—shapes climate discourse in African education (David, 2024). The study revealed that Western epistemologies in climate programs often *exclude or tokenize Indigenous perspectives*. For instance, climate curricula tended to present scientific facts and technological fixes while sidelining local knowledge about weather patterns or community resilience practices. This marginalization of Indigenous Knowledge Systems was traced to a colonial legacy in which Western education deemed local wisdom as “unscientific.” By undervaluing Africa's rich ecological knowledge (such as farmers' observations or pastoralists' adaptive strategies), climate psychology interventions—like public awareness campaigns or school programs—became less effective for local communities.

Cross-cultural studies further highlight the bias of Western assumptions. In a study spanning 63 countries—the largest climate change psychology experiment to date—researchers noted significant variation in what motivates climate action across cultures (Doell et al., 2024). Western interventions based on raising abstract risk awareness or individual guilt did not universally translate into action in non-Western contexts. Similarly, research on climate change inaction in China (Xiang et al., 2019) demonstrated that Western-oriented individualism correlates with lower willingness to act, whereas collectivist values common in East Asian contexts led to more proactive behavior. This finding suggests that Western psychology's focus on individual responsibility might misjudge or under-appreciate the power of collective efficacy found in other cultures. Another example is the concept of eco-anxiety—fear and worry about climate change—which has gained prominence through studies largely on Western youth. Pacific Island scholars have pointed out that Western notions like “climate anxiety” need reframing to fit Indigenous Pacific worldviews, where climate distress may be expressed through community narratives or spiritual terms rather than individual clinical anxiety (Newport et al., 2024). The risk of imposing Western diagnostic labels on non-Western experiences is that researchers might misinterpret resilience and coping mechanisms that don't fit Western mental health categories.

The publication and authorship patterns in climate psychology also serve as a case study in Western bias. A 2025 bibliometric review of climate change psychology literature found a heavy concentration of contributions from North America and Europe, with the Global South starkly underrepresented (Anjum and Aziz, 2025). It noted that even when research examined communities in Asia, Africa, or Latin America, it was often led by Western institutions with little involvement from local scholars. This imbalance means that the research questions, theoretical framing, and interpretations are filtered through Western lenses. Consequently, phenomena of great importance to non-Western populations—for example, the role of religion or traditional values in climate adaptation, or the mental health impacts of cultural loss due to environmental change—have been under-studied (“topic-blindness”) in the climate psychology literature (Usku et al., 2024). Collectively, these case studies demonstrate that Western epistemological bias is not an abstract concern but a tangible influence that shapes what we study, how we study it, and whose voices are heard in climate change psychology.

#### 1.2.4 Impact on climate change policy and action

The Western bias in climate psychology research does not stop at academia—it cascades into climate change policy and action strategies. Psychological research informs how we design public engagement campaigns, educational programs, and even international climate negotiations strategies. When that research is Western-centric, climate policies risk being less inclusive, less effective, and less just on a global scale. One major impact is the tendency to favor solutions aligning with Western technocratic thinking. Global climate policy has often emphasized technological innovation (e.g., renewable energy tech and carbon capture) and market-based mechanisms (like carbon trading) as panaceas. These approaches reflect Western modernist faith in technology and economics, sometimes at the expense of social or cultural dimensions. A decolonial analysis of the recent UN climate negotiations noted that current policy discourse remains rooted in “technocratic foundations,” presuming a continuation of the Western-developed systems that caused the crisis (Reed et al., 2024). As a result, important alternative perspectives—such as Indigenous approaches of living with and respecting the land—are sidelined. For example, although the Paris Agreement created a platform for Indigenous peoples, in practice Indigenous representatives have had limited ability to influence negotiations, often confined to observer roles with minimal input on decision texts (Reed et al., 2024). This exclusion means policies may ignore local psychological readiness, community values, and traditional practices that are crucial for successful implementation.

At the national and community level, excluding non-Western epistemologies can lead to climate action strategies that misfit local contexts. Western psychology-based campaigns often focus on individual behavior change (urging people to recycle, and drive less, etc.) and assume information deficits or cognitive biases are the main barriers to action. However, in many non-Western communities, collective action, structural issues, or issues of climate justice are more salient. If policy makers rely solely on Western psychological models, they might implement programs that fail to engage the public in, say, rural India or the Amazon because they overlook community decision-making structures or spiritual connections to the environment. Furthermore, ignoring Indigenous knowledge in adaptation planning can result in maladaptive outcomes. Indigenous communities have intimate knowledge of local ecosystems, for instance, understanding monsoon variability or using traditional burning to prevent wildfires—which is psychological as well as practical knowledge built over generations. Western-trained experts who discount this may impose solutions that locals find culturally alien or that simply don't work as well. Studies have shown that when climate initiatives marginalize or co-opt Indigenous knowledge into Western frameworks, they create “epistemic inequality” that ultimately limits resilience (David, 2024). For example, distributing high-tech weather alert systems in a region while ignoring elders' climate indicators and land-based education can erode trust and reduce community uptake of adaptation measures.

The bias also has implications for sustainability planning and climate justice. Western epistemologies traditionally prioritize human control over nature and economic growth, which can conflict with sustainable practices valued in other cultures. Policies influenced by Western psychology might emphasize public *perception* of climate risks or economic cost-benefit analyses (e.g., how to nudge consumers toward electric cars), whereas a more holistic approach might incorporate ethical relations with nature or duties to future generations—aspects highlighted in many Indigenous teachings. The exclusion of these perspectives can lead to sustainability plans that lack moral authority or grassroots support outside the West. In global climate negotiations, Western nations' psychological framing of climate responsibility (often focusing on current emissions and technical fixes) has clashed with the calls from developing nations to acknowledge historical injustices, loss of culture, and the rights of nature. Thus, Western-centric climate psychology can inadvertently support a narrow vision of climate action that doesn't fully address equity. As scholars argue, tackling climate change effectively demands an acknowledgment of the colonial legacies in knowledge systems (Mignolo, 2007), and a commitment to equity and justice in climate governance, ensuring that local and Indigenous knowledge inform adaptation and mitigation efforts. Failing to do so keeps climate policy less innovative and less fair, missing opportunities for solutions that are grounded in diverse human experiences with the natural world.

#### 1.2.5 Proposed solutions: integrating non-western epistemologies in climate psychology

Moving forward, climate change psychology can become more globally relevant and equitable by actively integrating non-Western epistemologies and methodologies. Figure 1 summarizes the proposed solutions. Below are key recommendations for researchers and policymakers to reduce Western bias and enrich the field.

##### 1.2.5.1 Embrace pluralistic theoretical frameworks

Researchers should adopt frameworks that value multiple worldviews. Approaches like *Two-Eyed Seeing* encourage using Western scientific insight and Indigenous wisdom together (Turner et al., 2024). This could mean jointly framing research questions through, say, both a cognitive-behavioral lens and an Indigenous relational lens. Incorporating concepts such as interconnectedness (common in Eastern and Indigenous philosophies) alongside Western theories can broaden understanding of climate-related behavior beyond individual cognition. For example, climate anxiety could be studied not only as an individual psychological state but also as a collective spiritual-ecological response, drawing on Indigenous concepts of ecological grief or balance.

##### 1.2.5.2 Expand methodological diversity

To overcome Western methodological limitations, climate psychology should integrate qualitative, participatory, and community-based methods on equal footing with quantitative approaches. Participatory Action Research and community-led studies allow local populations to guide research priorities and co-produce knowledge (Turner et al., 2024). Methods like storytelling, focus groups in local languages, and ethnography can capture nuances that standardized surveys miss. By using indigenous-sensitive methods—for instance, respecting oral histories and traditional ecological knowledge as valid data—researchers can ensure that findings are culturally grounded. Such methods were successful in identifying how Indigenous Tacana communities in Bolivia perceived climate changes differently yet complementarily to meteorologists (Bauer et al., 2022). Blending methodologies (mixed methods and intercultural comparisons) should become standard practice to validate results across cultural contexts.

##### 1.2.5.3 Increase inclusivity and collaboration

It is critical to decolonize the research process itself. This involves actively including scholars and knowledge holders from the Global South and Indigenous communities in climate psychology projects. Structural changes like equitable funding, diverse editorial boards, and North–South research partnerships are needed to amplify non-Western voices (Anjum and Aziz, 2024). Collaboration should go beyond using Indigenous communities as study subjects—they should be research designers, co-authors, and decision-makers. Empowering Indigenous youth through training in psychology research, for example, can bridge Western academic skills with traditional knowledge. Likewise, Western researchers must educate themselves on cultural protocols and avoid imposing their own frames. Ethical guidelines for cross-cultural research (obtaining community consent, reciprocity in benefits, and respecting intellectual property of traditional knowledge) are essential for trust-building. When scholars from different epistemic backgrounds work together as equals, the resulting climate psychology insights are more likely to be globally relevant and socially just.

##### 1.2.5.4 Inform policy with multicultural knowledge

Climate policies and communication strategies should be informed by this enriched, pluralistic psychological science. Practically, this means creating platforms for Indigenous and local knowledge in policy planning—for example, consulting tribal elders or local farmers when designing national adaptation programs, and incorporating their psychological coping strategies into official plans. Governments and NGOs can use culturally tailored messaging that resonates with communal values or spiritual beliefs rather than copying Western campaign models. International bodies like the IPCC have begun to recognize Indigenous knowledge as “complementary” to scientific evidence (Northwestern Buffett Institute for Global Affairs, 2021) and this should be operationalized by including indigenous psychologists or social scientists in drafting assessment reports. By integrating multiple epistemologies, climate interventions can appeal to a broader range of motivations (honor, stewardship, and sacred values) beyond the Western emphasis on personal risk and utility. The result would likely be more effective engagement and more robust policy outcomes, as strategies are co-developed with those who are most impacted and informed by non-Western experiences.

##### 1.2.5.5 Critical reflexivity and ongoing evaluation

Finally, the field must cultivate a habit of critical reflexivity—continually questioning whose knowledge is centered. Academic training in climate psychology should include exposure to Indigenous studies, cross-cultural psychology, and post-colonial critiques so that new researchers are aware of implicit biases. Journals and conferences can encourage authors to discuss the cultural scope and limits of their theories. Moreover, ongoing evaluation of interventions in diverse settings can highlight mismatches where Western-based programs falter, providing learning opportunities to adapt frameworks. By treating Western epistemology as one of many rather than the default, climate psychology can transform into a more inclusive science. This transformation aligns with broader calls to “rebalance reciprocal relationships with the natural world” in research and policy (Reed et al., 2024), essentially merging scientific understanding with the wisdom of living sustainably that many non-Western cultures have preserved.

## 2 Conclusion

Western epistemologies have undeniably shaped the young field of climate change psychology, from the theories we build to the methods we deploy and the policies we inform. This Western-centric foundation has yielded insights into cognitive biases, risk perceptions, and behavior change, but it also imposes limitations by neglecting the rich diversity of human thought and experience related to our changing climate. The biases toward individualism, human-nature separation, and quantitative empiricism mean that climate psychology, as it stands, does not fully represent *universal* psychology—it represents a Western-adapted psychology applied to a global problem. The implications are far-reaching: research findings may not generalize, well-intentioned interventions may misfire outside Western contexts, and global climate solutions may fail to account for those most affected and knowledgeable. Correcting this bias is not about discarding Western science, but about expanding the knowledge project to be more inclusive and just. Integrating non-Western epistemologies—Indigenous knowledge systems, collectivist values, experiential and spiritual understandings—will enhance the scientific robustness of climate psychology and its relevance for all peoples. It will help create climate action strategies that are culturally resonant and empower communities rather than treating them as passive recipients of Western expertise. In essence, embracing epistemic diversity in climate change psychology is both an ethical mandate and a practical necessity for addressing the climate crisis. By decolonizing research practices and co-creating knowledge across cultures, we can better understand the psychological dimensions of climate change and foster a truly global response that draws on *all* ways of knowing to sustain our shared planet.

## Data Availability

The original contributions presented in the study are included in the article/supplementary material, further inquiries can be directed to the corresponding author.
